# Magnetic Domain-Wall Induced Electric Polarization in NdCrO_3_ Polycrystalline Ceramic

**DOI:** 10.3390/ma13081904

**Published:** 2020-04-17

**Authors:** Songwei Wang, Yang Bai, Xin Zhang, Liguo Fan, Huaiying Zhou

**Affiliations:** 1 School of Materials Science and Engineering, Guilin University of Electronic Technology, Guilin 541004, China; swwang666@126.com (S.W.); yangbai19940711@163.com (Y.B.); 15803462571@163.com (L.F.); zhy@guet.edu.cn (H.Z.); 2 Guangxi Key Laboratory of Information Materials, Guilin University of Electronic Technology, Guilin 541004, China

**Keywords:** NdCrO_3_ ceramics, magnetic property, magnetoelectric properties

## Abstract

We reported the magnetic, dielectric and magnetoelectric properties of NdCrO_3_ polycrystalline ceramics. Magnetization curves revealed two magnetic transitions at 227 K and 38 K, which corresponded to Cr^3+^ canted antiferromagnetic ordering and Cr^3+^ spin reorientation phase transition, respectively. At 11.5 K, a Schottky-type anomaly was observed, caused by Nd^3+^ ground doublet Zeeman splitting. High-temperature dielectric relaxation exhibited a type of thermally activated relaxation process, which mainly resulted from the Maxwell–Wagner effect. The spin-reorientation of Cr^3+^ ions and the Nd^3+^ ground doublet splitting were observed to be accompanied by an electric polarization. The polarization could be induced by the presence of the antiferromagnetic-type domain walls, which led to spatial inversion symmetry breaking.

## 1. Introduction

Multiferroic materials, defined as those with multiple order parameters coexisting and coupling with each other, have received great attention in recent years due to their potential applications in multiple-state memory elements and spintronic devices [1,2]. However, single-phase multiferroics are rare because ferroelectricity and magnetism are often mutually exclusive [3]. Therefore, an exploring of multiferroics based on the emerging physical mechanism is an important and challenging issue. Until now, several mechanisms have been established for magnetically induced ferroelectricity, such as metal–ligand *p*–*d* hybridization, symmetric-exchange striction, and antisymmetric inverse Dzyaloshinskii–Moriya interaction [4,5,6]. In recent years, the polarization induced by the magnetic domain wall has been demonstrated theoretically, such as those occurring in Lu_2_CoMno_6_ [7], R_2_NiMno_6_ [8] and AFeO_3_ (A = Lu, Y, Gd, Sm) [9,10]. 

Rare-earth orthochromites (RCrO_3_) represent an attractive class of compounds due to the cross coupling between the 3d spins of transition metal ions and 4f moments of rare-earth ions, similar to RMnO_3_. The Néel temperature can be improved without affecting the saturation magnetization when Mn is substituted with Cr [11,12]. RCrO_3_ (R = La, Pr, Sm, Nd, Gd, Tb, Ho, Dy, Er, Tm, Lu) compounds have shown potential multiferroic properties mostly due to the exchange striction between the R and Cr moments [13,14,15,16,17,18,19,20,21]. The NdCrO_3_ magnetization curve differs significantly from those typically found in the rare-earth orthochromites [22]. Below the Néel temperature (227 K), two peaks were observed at about 200 K and 35 K, respectively. In addition, NdCrO_3_ is with rich ferroelectric property. Indra et al. [19] reported ferroelectricity around ∼88 K in NdCrO_3_, and the synchrotron diffraction study suggested that the ferroelectricity arose from the structural transformation from the centrosymmetric space group Pnma to a non-centrosymmetric space group Pna2_1_. Shanker et al. [21] reported that NdCrO_3_ is a relaxor ferroelectric (500 K at 1 MHz). Yin et al. [20] reported a dielectric anomaly in the vicinity of the Nd^3+^ spins ordering the temperature of the NdCrO_3_, but the nature of the anomaly was not clear. 

In this work, we studied the magnetic, dielectric and magnetoelectric properties of a NdCrO_3_ polycrystalline sample in the temperature range of 5 K–360 K. We observed interesting novel phenomena such as the dielectric anomaly at reorientation temperature (~38 K) of Cr^3+^, which could be affected by the magnetic field and may indicate an electric polarization. The magnetic field-dependent polarization could be induced by the presence of anti-phase antiferromagnetic (AFM)-type domain walls, which leads to spatial inversion symmetry breaking [9].

## 2. Experiment

NdCrO_3_ polycrystalline ceramics were synthesized by conventional solid-state reaction. The detailed preparation process was similar to our previous work [23]. X-ray diffraction (XRD) patterns of the samples were recorded using a PANalytical X^’^pert Pro PW 3040 diffractometer with Cu Kα radiation. The morphology of the samples was measured by a field emission scanning electron microscope (FESEM). The dielectric property of the samples was measured by a Precision Impedance Analyzer (Wayne Kerr Electronics, 6500B, Cavendish Square, London, England). A physical properties measurement system (PPMS 9T, Quantum Design) was used for measuring the temperature dependence of the magnetization and dielectric constants of the samples.

## 3. Results and Discussion

**Figure 1:**
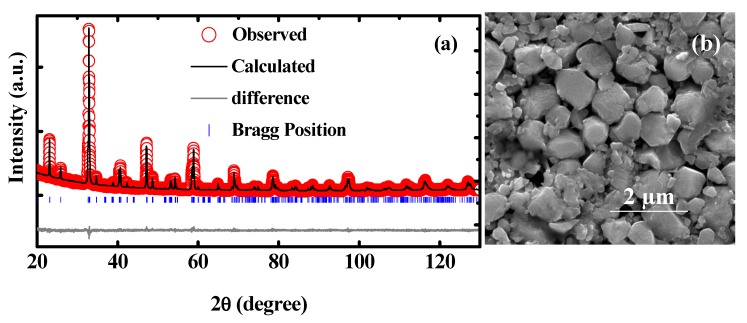
(Color online) (**a**) Rietveld refinement result of the XRD pattern, (**b**) FESEM morphology of the NdCrO_3_ sample.

Figure 1a shows the Rietveld refinement result of the XRD data of the NdCrO_3_ polycrystalline ceramics. The Rietveld refinement with space group *Pbnm* delivers the lattice parameters of a = 5.420(9) Å, b = 5.483(3) Å, and c = 7.693(3) Å for the compound with a goodness-of-fitting value of χ^2^ = 1.53 and *Rwp* = 14.6%. The FESEM image of the NdCrO_3_ bulk is shown in Figure 1b. The morphology of the sample consisted of irregular grains of different sizes, in which large grains were formed by stacking the thin plate layer by layer.

**Figure 2:**
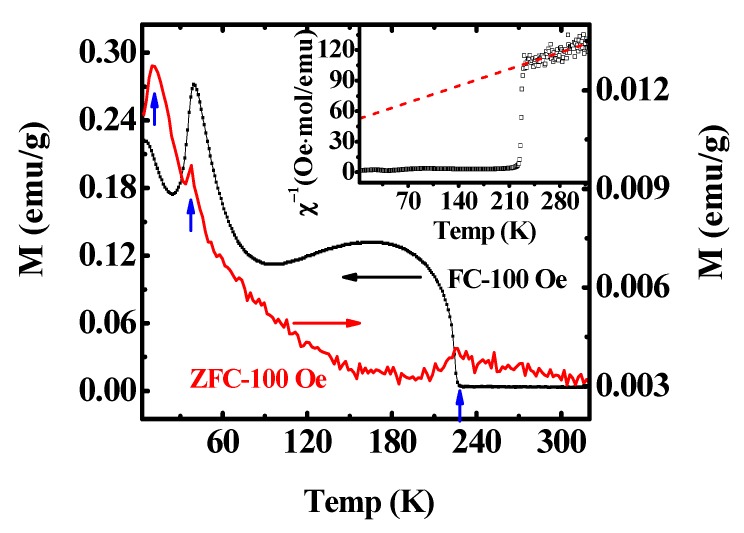
(Color online) Temperature-dependent magnetization curve of NdCrO_3_ at the magnetic field of 100 Oe. The inset shows the reciprocal of the dc magnetic susceptibility vs. the temperature.

Figure 2 shows the zero-field-cooled (ZFC) and field-cooled (FC) temperature-dependent dc magnetization from 5 K to 320 K under a measuring field of 100 Oe. Two magnetic transitions, T_N_ and T_SR_, occurred at about 227 K and 38 K, respectively, which were consistent with previous studies [22,24,25,26] and their magnetic behavior could be explained by the strong effective field on the Nd^3+^ moments exerted by the ordered Cr^3+^ spin system [27,28]. The first transition temperature T_N_ (227 K) corresponded to canted AFM transition of the Cr^3+^ magnetic moments and the system showed a weak ferromagnetism. Thus, below T_N_, the Cr^3+^ moments configuration was from Γ_2_(F_x_, C_y_, G_z_) into Γ_1_(A_x_, G_y_, C_z_) at 38 K (spin reorientation) due to the magnetic crystal anisotropy. From the ZFC curves in Figure 2, a small hump was observed at T ≈ 11.5 K (named as T_SP_), which originated from the two-level Schottky effect caused by the split of the ‘Nd’ ground doublet [29]. The inset showed the reciprocal of the dc magnetic susceptibility curve (FC) and obeyed the Curie–Weiss law. Furthermore, the derived effective magnetic moment was ~5.845 μ_B_/f.u., which was bigger than the theoretical one (5.3 μ_B_/f.u.). The difference was either due to the magnetic polarization of the Nd moments by the Cr moments or ascribed to a slight change of the susceptibility due to the crystal field splitting effects of the excited ^7^F*_J_* levels [23].

**Figure 3:**
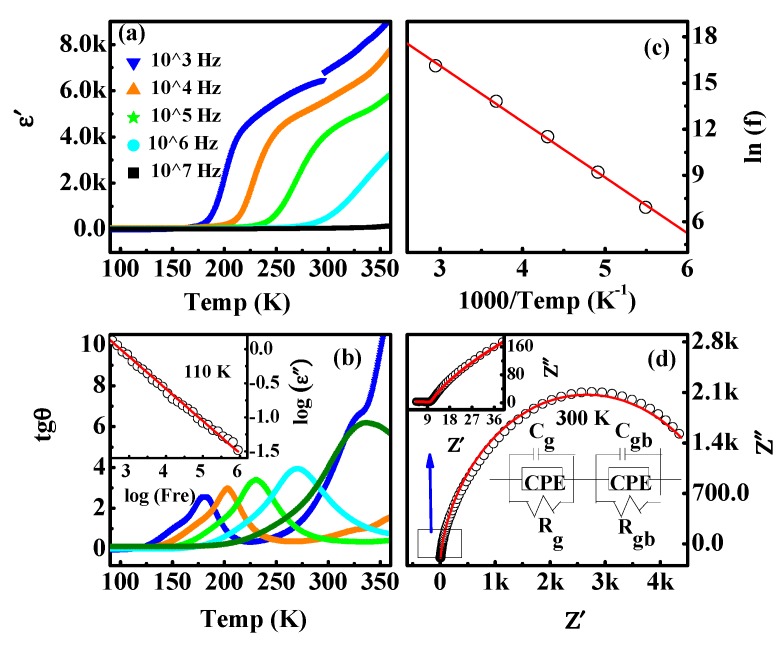
(Color online) (**a**) The real part (*ε′*) of the dielectric constant vs. temperature at various frequencies. (**b**) Corresponding dielectric loss (tan δ). The inset is log (*ε″*) vs. log (f) at 110 K in the low frequency region and the solid line is the one with slope −1. (**c**) The lnƒ vs. 1000/T derived from tan δ. (**d**) Nyquist plots at 300 K for the circuit shown.

Frequency dependence of the real part (*ε′*) of the dielectric constant (Figure 3a) and the loss (tan δ) (Figure 3b) from 100 K to 360 K exhibited a thermal-activated relaxation behavior [23]. In the frequency range probed, dielectric relaxations could arise from two mechanisms: (a) the mobility of oxygen vacancies. The activation energy in various perovskite materials was in the range of 0.9–1.48 eV for the dielectric relaxations related to the mobility of oxygen vacancy [30]. In general, for a thermally activated relaxation process, the variation of peak position (T_M_) can be described by the Arrhenius law:

Ƒ = ƒ_0_ exp (-*E_a_***/***k_B_T_M_*) (1)

where ƒ_0_ is the pre-exponential, *E_a_* is the activation energy. Figure 3c shows the Arrhenius plots of tan δ for the relaxation. The solid line shows the fitting to the experiment data by Equation (1). The activation energy was derived to be about 0.313 eV. Therefore, the type of dielectric relaxation was not attributed to the mobility of oxygen vacancies. (b) Maxwell–Wagner relaxation originating from different conductivities between regions in the sample [31]. The characteristic of the Maxwell–Wagner relaxation mechanism is the f^−1^ dependence of the imaginary part of dielectric constant data (*ε″*) at lower frequencies [23,31]. The slope of log(*ε″*) vs. log(f) is about (−1) in the frequency range of 10^2^–10^6^ Hz, as shown in the inset of Figure 3b at 110 K, clearly suggesting the presence of the Maxwell–Wagner relaxation. Since the contributions arising from grain (g), grain boundary (g_b_), and specimen-electrode interface can be separated by Nyquist plots [32], the Nyquist plot (Z” vs. Z’) was studied (shown in Figure 3d). The Nyquist plot was well fitted with an equivalent circuit. The circuit made up of two sub-circuits in the series indicated that there were two relaxations corresponding to the grain boundaries and grains [32,33].

**Figure 4:**
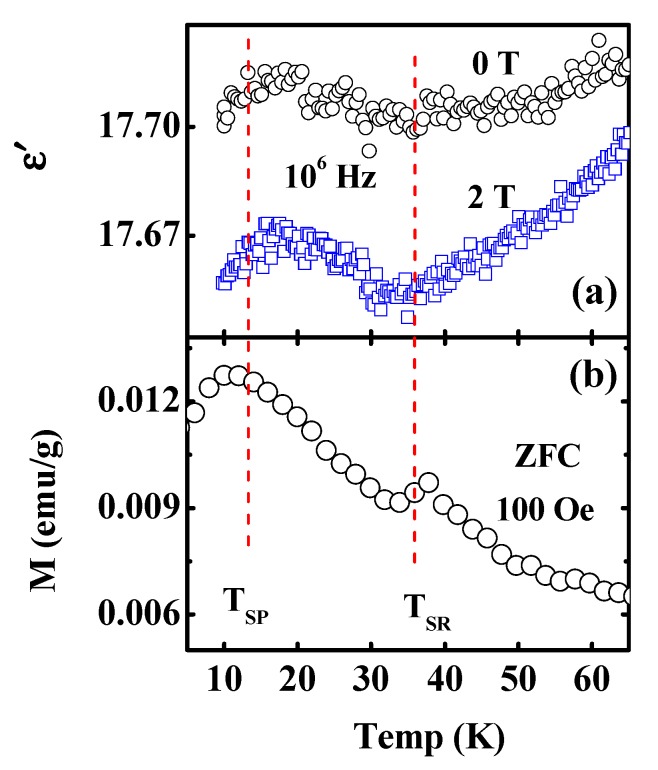
(Color online) (**a**) *ε′* vs. temperature at different fields. (**b**) ZFC magnetization curve under at 100 Oe.

Figure 4a shows the dielectric behavior at a high frequency (10^6^ Hz) in 5 K–65 K at zero and 2 T fields. At a zero magnetic field, there were dielectric anomalies around T_SR_ and T_SP_ marked in Figure 4b and the behaviors were more significant when an external magnetic field was applied. The results indicated that there are native magnetoelectric coupling. As mentioned before, the magnetic configuration was changed from Γ_2_(F_x_, C_y_, G_z_) to Γ_1_(A_x_, G_y_, C_z_) at 38 K. Γ_1_(A_x_, G_y_, C_z_) configuration is an AFM-type magnetic structure. Therefore, the electric polarization does not arise from the non-collinear magnetism. Yanez-Vilar [7] reported that a polarization may arise as a result of anti-phase AFM domain boundaries, i.e., it does not require non-collinear magnetism or spin-orbit coupling. In addition, Yang reported that an improper polarization originates from an exchange striction mechanism that drives a polar displacement of the oxygen ions located at the magnetic domain walls in SmFeO_3_ with an orthorhombic structure [9]. Moreover, additional calculations ratified that the mechanism was general among magnetic perovskites with an orthorhombic SmFeO_3_-like structure. Therefore, we may elucidate the dielectric anomaly by an AFM-type spin arrangement at domain walls that break the spatial inversion symmetry and cause polarization along a specific direction. Such an explanation agrees well with the theoretical calculations for the NdCrO_3_ material [9].

## 4. Conclusions

In summary, the magnetic, dielectric and magnetoelectric properties of polycrystalline NdCrO_3_ ceramics were studied. Magnetization measurements showed that there were three magnetic transitions at 227 K, 38 K and 11.5 K, respectively. The dielectric constant measurements showed that the high temperature relaxation results from the Maxwell–Wagner effect. The abnormal dielectric phenomena at 10^6^ Hz at a low temperature indicated that there was native magnetoelectric coupling. Further analysis showed that the magnetic field-dependent polarization can be induced by the presence of the AFM-type spin arrangement domain walls, which leads to spatial inversion symmetry breaking.
